# Machine learning improves our knowledge about miRNA functions towards plant abiotic stresses

**DOI:** 10.1038/s41598-020-59981-6

**Published:** 2020-02-20

**Authors:** Keyvan Asefpour Vakilian

**Affiliations:** 1 Department of Agrotechnology, College of Abouraihan, University of Tehran, Tehran, Iran; 2 Private Laboratory of Biosensor Applications, Hamadan, Iran

**Keywords:** Biochemistry, Plant sciences

## Abstract

During the last two decades, human has increased his knowledge about the role of miRNAs and their target genes in plant stress response. Biotic and abiotic stresses result in simultaneous tissue-specific up/down-regulation of several miRNAs. In this study, for the first time, feature selection algorithms have been used to investigate the contribution of individual plant miRNAs in *Arabidopsis thaliana* response towards different levels of several abiotic stresses including drought, salinity, cold, and heat. Results of information theory-based feature selection revealed that miRNA-169, miRNA-159, miRNA-396, and miRNA-393 had the highest contributions to plant response towards drought, salinity, cold, and heat, respectively. Furthermore, regression models, i.e., decision tree (DT), support vector machines (SVMs), and Naïve Bayes (NB) were used to predict the plant stress by having the plant miRNAs’ concentration. SVM with Gaussian kernel was capable of predicting plant stress (*R*^2^ = 0.96) considering miRNA concentrations as input features. Findings of this study prove the performance of machine learning as a promising tool to investigate some aspects of miRNAs’ contribution to plant stress responses that have been undiscovered until today.

## Introduction

microRNAs (miRNAs) are small single-stranded RNAs with low protein-coding potential^1^. Although plant miRNAs target only a small number of mRNAs (<1%)^2^, the role of miRNA-controlled gene regulation in plants cannot be neglected because most of the target mRNAs participate in most plant developmental processes^3,4^. Furthermore, there are evidences showing the relationships between plant stress responses and changes in miRNAs’ expression^5,6^. miRNAs are known as negative post-transcription regulators since they exert specific binding to their target mRNAs or repressing target mRNA translation^7–9^.

Among the major plant abiotic stress sources, drought, salinity, cold, heat, ultraviolet irradiation, carbon dioxide, and heavy metal pollution have significant effects on plant morphological, physiological, and biochemical characteristics^10,11^. To adapt and survive under stress conditions, plants exert miRNA up/down-regulation which results in gene expression reprogramming to restore cellular homeostasis^12,13^. Plant miRNA expression towards stress is generally spatial (plant tissue) and temporal (developmental/growth stage) specific^4,14^.

With the identification of stress-responsive miRNAs, useful information on their role in improving the stress tolerance mechanism of plants can be obtained. A search in bibliographic resources reveals that hundreds of research studies have been dedicated to the changes in plant miRNA expression in response to biotic and abiotic stresses. A large part of these studies has focused on *Arabidopsis thaliana*, *Brachypodium distachyon*, *Glycine max*, *Hordeum vulgare*, *Medicago truncatula*, *Manihot esculenta*, *Phaseolus vulgaris*, *Populus euphratica*, *Populus trichocarpa*, *Populus tremula*, *Triticum turgidum*, *Oryza sativa*, *Vigna unguiculate*, and *Zea mays*^15^. Studies have shown that a miRNA most probably functions in several stresses in one hand. miRNA-167, miRNA-169, miRNA-171, miRNA-319, miRNA-393, miRNA-394, and miRNA-396 are some examples of miRNAs that function in many abiotic stress-related processes^16–20^. On the other hand, a stress can involve changes in the expression of several miRNAs. As an example, nitrogen deficiency can result in an overexpression of RNA-156, miRNA-160, miRNA-171, miRNA-780, miRNA-826, miRNA-842, and miRNA-846^1^.

Some of plant abiotic stresses such as drought, salinity, cold, and heat are of major constraints to agricultural productivity worldwide^21^. The study of miRNAs involving these stresses and their contribution to plant response is important since it can provide us with valuable information on plant stress physiology. However, only involved miRNAs, their expression in stress conditions, and their target genes are already identified in previous studies and their contribution to plant response towards different levels of plant stress is still a matter of question. Therefore, it seems that investigating the contribution (in other words, importance) of each miRNA in plant stress response can be interesting. The preparation of a database based on the observations of miRNA expressions at different levels of plant stress can be the first step. Methods such as northern blot and polymerase chain reaction (PCR) which have been widely used to measure miRNA expressions suffer from weak analytical characteristics, e.g., limit of detection, response linear range, and precision^22^. Therefore, it seems that using biosensors equipped with gold nanoparticles which work on the basis of nanoparticle aggregation is a reliable method to gather required information for such databases^23,24^. Afterwards, using feature selection algorithms to rank the miRNAs will be a possible solution in miRNA contribution investigations.

Feature selection is one of the fundamental problems in the fields of machine learning and pattern recognition^25,26^. Several approaches have been employed in feature selection, such as: embedded^27^, wrapper^28^, and filter^29^ methods. These methods use various evaluation criteria for scoring the input features. Among these criteria, information theory-based measurements achieve excellent performance according to their robust algorithm^30^. In contrast with conventional feature selection methods which discard features that are highly correlated to other features but relevant to the target class^31^, information-based feature selecting methods such as cooperative game theory and minimum redundancy - maximum relevance do not ignore features which have strong discriminatory power as a group but are weak as individuals^32^. In cooperative game theory, features that make a big difference as group are usually selected. However, it is possible that they individually perform poorly^33^. In fact, the accuracy of target prediction is assumed as a game in which the features (miRNAs in this study) are the players and a team of players should be selected who can achieve the best results (better prediction of plant stress). In this method, a score is assigned to each feature and the features which are identified with low scores can be eliminated in the measurements.

Machine learning can also be used to predict plant stress by having the plant miRNA expressions. In this situation, machine learns the complex non-linear patterns between inputs (miRNA expressions) and output (plant stress) using the training data in the database and predicts the stress condition of unknown plant samples. Although several learning algorithms including supervised, unsupervised, reinforcement, sparse dictionary, and rule-based learnings have been extensively utilized in previous studies, supervised learning is a reliable and efficient method for life science problems^34,35^. Decision tree (DT), support vector machines (SVMs), least-square support vector machines (LSSVMs), and Naïve Bayes (NB) can be used as supervised learning methods to find the patterns in a database^36,37^. Acceptable performance of the machine (which is indicated by performance evaluation criteria) will show that the expressions of the selected miRNAs which have been used to train the machine are the most important miRNAs in plant response towards stress.

The objectives of this study are: (a) to measure the effects of different levels of abiotic stress including drought, salinity, cold, and heat on the expression of already-known *Arabidopsis thaliana* miRNAs using a gold nanoparticle-based optical biosensor; (b) to investigate the contribution of miRNAs to the plant response towards studied abiotic stresses using information theory-based feature selection algorithms; and (c) to use supervised regression models to predict the plant stress by having the plant miRNA expressions.

## Results and Discussion

**Figure 1:**
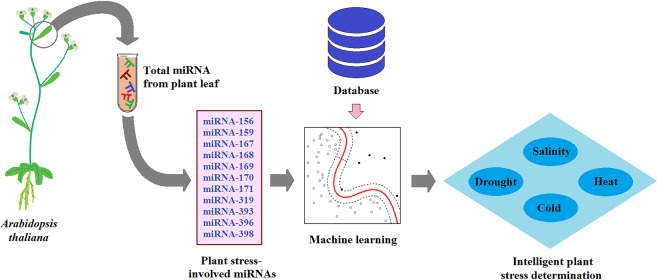
The proposed model to link the plant miRNA concentration to the stress using machine learning.

Several studies have shown that 11 miRNAs exert tissue-specific expression towards major abiotic stresses in *Arabidopsis thaliana*^1^. This study tries to demonstrate a model in which, machine learning links the plant leaf miRNA expression to the stress. In this situation, a successful learning-based model will be capable of precise plant stress determination by having the concentration of stress-involved miRNAs. Figure 1 depicts the model proposed in this study. Furthermore, features selection algorithms can reveal the contribution of each miRNA to the plant stresses; the information which may require rather complex and expensive laboratory efforts to obtain.

### The effects of different levels of abiotic stress on miRNA concentrations

The miRNA concentrations towards different levels of abiotic stress is brought in Table 1. This information was necessary to construct the database required for machine learning algorithms. Similar to the results obtained in previous investigations, Table 1 shows that the studied miRNAs were significantly affected by the plant major abiotic stresses. The miRNA concentration determination was carried out using a gold nanoparticles-based optical biosensor because the biosensor response towards analyte is generally more sensitive and specific than that obtained by conventional analytical methods, e.g., qRT-PCR, northern blot, and microarrays^22,38,39^. Although an optical biosensor is developed in this study, amperometric^40^, impedimetric^41^, and fluorescence-based^42^ biosensors are also introduced for miRNA measurements. However, on one side, electrochemical methods require extensive electrode pre-treatments and costly equipment. On the other side, fluorescence-based methods are not sensitive which decreases their application as a promising technique for miRNA determination^43^. A signal-to-noise ratio of 3:1 was considered to calculate the limit of detection (LoD) of the developed biosensor in this study^44^. It was found that the LoD of the biosensor is equal to 0.5 fM. As another important analytical parameter, the resolution of the biosensor was 1 fM.

**Table 1:** Plant leaf miRNA concentrations (fM) under stress conditions. Treatments are demonstrated in the text.

Treatment	miRNA concentration										
	miRNA-156	miRNA-159	miRNA-167	miRNA-168	miRNA-169	miRNA-170	miRNA-171	miRNA-319	miRNA-393	miRNA-396	miRNA-398
Control	125 ± 6	45 ± 3	79 ± 7	16 ± 4	84 ± 6	322 ± 15	8 ± 1	578 ± 6	65 ± 6	844 ± 29	13 ± 3
*W*_1_	462 ± 23 ^c^	74 ± 4 ^d^	545 ± 28 ^d^	25 ± 4 ^d^	63 ± 4 ^a^	365 ± 21 ^d^	13 ± 2 ^d^	594 ± 9 ^d^	83 ± 5 ^d^	1342 ± 18 ^d^	14 ± 4 ^a^
*W*_2_	670 ± 17 ^b^	115 ± 7 ^c^	963 ± 47 ^c^	41 ± 4 ^c^	61 ± 5 ^a^	392 ± 17 ^c^	19 ± 2 ^c^	612 ± 10 ^c^	102 ± 9 ^c^	1526 ± 17 ^c^	12 ± 2 ^a^
*W*_3_	831 ± 25 ^a^	143 ± 11 ^b^	1328 ± 42 ^b^	64 ± 6 ^b^	45 ± 3 ^b^	440 ± 23 ^b^	25 ± 3 ^b^	625 ± 8 ^b^	136 ± 8 ^b^	1942 ± 25 ^b^	12 ± 3 ^a^
*W*_4_	843 ± 32 ^a^	234 ± 14 ^a^	1743 ± 53 ^a^	93 ± 8 ^a^	29 ± 4 ^c^	487 ± 20 ^a^	37 ± 4 ^a^	647 ± 8 ^a^	175 ± 11 ^a^	2416 ± 32 ^a^	14 ± 3 ^a^
*S*_1_	174 ± 10 ^d^	94 ± 8 ^c^	1005 ± 65 ^c^	44 ± 4 ^c^	124 ± 8 ^d^	349 ± 21 ^c^	15 ± 2 ^b^	603 ± 7 ^c^	97 ± 4 ^d^	974 ± 15 ^d^	15 ± 3 ^a^
*S*_2_	301 ± 21 ^c^	263 ± 17 ^b^	1655 ± 85 ^b^	57 ± 4 ^b^	194 ± 9 ^c^	363 ± 11 ^b^	21 ± 2 ^a^	600 ± 10 ^c^	132 ± 8 ^c^	1081 ± 13 ^c^	11 ± 2 ^b^
*S*_3_	372 ± 22 ^b^	554 ± 28 ^a^	1664 ± 93 ^b^	83 ± 7 ^a^	247 ± 11 ^b^	361 ± 14 ^b^	20 ± 3 ^a^	671 ± 9 ^b^	176 ± 7 ^b^	1211 ± 19 ^b^	10 ± 3 ^b^
*S*_4_	435 ± 25 ^a^	546 ± 22 ^a^	2155 ± 53 ^a^	81 ± 5 ^a^	283 ± 10 ^a^	401 ± 14 ^a^	21 ± 4 ^a^	748 ± 11 ^a^	213 ± 9 ^a^	1307 ± 21 ^a^	7 ± 2 ^c^
*C*_1_	132 ± 8 ^a^	45 ± 3 ^a^	73 ± 6 ^a^	15 ± 4 ^d^	98 ± 8 ^d^	338 ± 21 ^c^	14 ± 2 ^d^	734 ± 8 ^d^	79 ± 5 ^c^	1138 ± 23 ^c^	13 ± 4 ^a^
*C*_2_	129 ± 7 ^a^	43 ± 2 ^a^	78 ± 8 ^a^	23 ± 3 ^c^	137 ± 8 ^c^	335 ± 11 ^c^	23 ± 2 ^c^	957 ± 11 ^c^	93 ± 4 ^b^	1472 ± 19 ^b^	13 ± 4 ^a^
*C*_3_	122 ± 10 ^a^	45 ± 3 ^a^	75 ± 9 ^a^	35 ± 5 ^b^	199 ± 9 ^b^	378 ± 14 ^b^	35 ± 3 ^b^	1143 ± 9 ^b^	124 ± 7 ^a^	2131 ± 30 ^a^	9 ± 2 ^b^
*C*_4_	126 ± 8 ^a^	46 ± 3 ^a^	71 ± 8 ^a^	52 ± 5 ^a^	244 ± 8 ^a^	422 ± 14 ^a^	45 ± 4 ^a^	1404 ± 9 ^a^	121 ± 9 ^a^	2142 ± 27 ^a^	3 ± 3 ^c^
*H*_1_	273 ± 22 ^d^	112 ± 11 ^c^	238 ± 23 ^d^	17 ± 2 ^a^	57 ± 4 ^a^	327 ± 17 ^a^	12 ± 1 ^d^	585 ± 7 ^d^	83 ± 4 ^d^	1005 ± 18 ^d^	13 ± 2 ^a^
*H*_2_	429 ± 27 ^c^	218 ± 21 ^b^	524 ± 33 ^c^	15 ± 3 ^a^	32 ± 5 ^b^	319 ± 25 ^a^	37 ± 1 ^c^	607 ± 9 ^c^	91 ± 4 ^c^	1321 ± 23 ^c^	12 ± 3 ^a^
*H*_3_	526 ± 28 ^b^	233 ± 15 ^b^	745 ± 30 ^b^	16 ± 4 ^a^	28 ± 6 ^b^	328 ± 18 ^a^	55 ± 5 ^b^	620 ± 7 ^b^	102 ± 10 ^b^	1584 ± 22 ^b^	14 ± 4 ^a^
*H*_4_	641 ± 31 ^a^	342 ± 14 ^a^	1254 ± 42 ^a^	15 ± 3 ^a^	19 ± 5 ^c^	325 ± 21 ^a^	94 ± 7 ^a^	642 ± 8 ^a^	117 ± 10 ^a^	1859 ± 24 ^a^	12 ± 4 ^a^

Data are shown as means ± SD (*n* = 5).

Different small letter within the same column for each treatment indicate significant differences between the stress levels based on LSD test (*P* < 0.01).

As shown in Table 1, some miRNAs have been induced during the stress condition whilst some other miRNAs have been inhibited or unaltered. As expected, the concentration of studied miRNAs has been changed towards at least one stress. The stress levels in this study are selected in a manner to divulge mild, moderate and severe stress conditions in the *Arabidopsis thaliana* plants. According to the table, some miRNAs, i.e., miRNA-171 (with concentrations lower than 100 fM even in induced form) and miRNA-398 (with concentrations lower than 20 fM) were found in very small amounts during the experiments which were induced and inhibited during the stress conditions, respectively. The small concentrations of these two miRNAs should not be interpreted their weak role in plant physiology and biochemistry. The target genes of miRNA-171 in *Arabidopsis thaliana* are *SCL6-II* and *SCL6*, the genes that function in plant root and leaf development, photochrome signalling, lateral organ polarity, meristem formation, vascular development, and stress response^45–47^. miRNA-398 is also an important miRNA which targets *CSD*, *COC5b-1*, and *CCS1* genes playing remarkable roles in Cu homeostasis, heavy metal tolerance, and oxidative stress^48^. There are several stress conditions that the studied miRNAs did not show any significant alteration towards them (*P* < 0.01) (Table 1): miRNA-398 in drought, miRNA-156, miRNA-159, and miRNA-167 in cold, and miRNA-168, miRNA-170, and miRNA-398 in heat conditions.

Although similar results have been reported in previous studies^1^, they have generally considered the effects of severe stress conditions. However, findings of this study revealed that even slight to moderate stress can significantly affect the miRNA concentration. This is interesting since the changes in miRNA concentration detected by an optical biosensor similar to the biosensor developed in this study can be useful for early detection of stress. In this situation, we should know that the result is not specific to a certain stress because as shown in Table 1, a miRNA can be affected be several stress sources.

According to the results, miRNA-167 exerted the highest change in its concentration towards stress among the studied miRNAs. A ca. 27-fold increment in this miRNA was observed during severe salinity stress. The function of miRNA-167 was not very clear in plant stress response until it was recently showed that the transgenic tomato plants overexpressing miRNA-167 exhibited reductions in leaf size and internode length as well as shortened petals, stamens, and styles^49^. In another study, the differential expression of the cassava miRNA-167 target genes (*MesARF6/8*) were observed to be associated with changes in the leaf shape and stomatal closure towards drought stress^50^.

### The contribution of miRNAs to the plant response towards abiotic stresses

The mean values of miRNA concentrations towards different levels of plant stress were used to construct a database suitable for machine learning. Table 2 shows the contribution of each miRNA to plant stress response which was obtained by implementing the feature selection algorithm on the constructed database. The concept of miRNA’s contribution (and importance) to a plant stress has been introduced in this study for the first time. It shows the possibility of proper prediction of a plant stress by having the concentration of a specific miRNA. As shown in Table 2, miRNA-169 concentration had the highest contribution to drought stress. This reveals that among the abiotic stress-involved miRNAs, drought condition has the highest correlation with miRNA-169 concentration in *Arabidopsis thaliana*. Accordingly, miRNA-159, miRNA-396, and miRNA-393 had the highest contributions to the salinity, cold, and heat stresses, respectively. The five most important miRNAs in each stress condition are shown in bold. Studies have shown that even large-scale data in standard databases can be classified with acceptable performance having the five features with the highest scores^32^.

**Table 2:** The importance of miRNAs in plant stress response. The numbers show the importance of the miRNAs. The lower the number, the higher the importance. The five most important miRNAs in each stress condition are shown in bold whilst the five least important miRNAs are in italic.

miRNA	Stress			
	Drought	Salinity	Cold	Heat
miRNA-156	**2**	*7*	*9*	**3**
miRNA-159	*10*	**1**	*11*	*8*
miRNA-167	*7*	*9*	*10*	6
miRNA-168	6	6	**5**	*9*
miRNA-169	**1**	**5**	**2**	*7*
miRNA-170	**3**	*10*	*7*	*11*
miRNA-171	*8*	*11*	**4**	**5**
miRNA-319	*9*	**4**	6	**4**
miRNA-393	**5**	**3**	*8*	**1**
miRNA-396	**4**	**2**	**1**	**2**
miRNA-398	*11*	*8*	**3**	*10*

According to Table 2, miRNA-169, miRNA-393, and miRNA-396 have important contributions to at least three studied abiotic stresses. This means that they are good indicators of a wide range of plant stress: from slight to severe stress conditions. These miRNAs have been extensively investigated in plant physiology since there are several evidences of significant changes in their expression towards stress not only in *Arabidopsis thaliana*, but in many plants such as *Phaseolus vulgaris*, *Populus euphratica*, *Populus trichocarpa*, *Populus tremula*, *Triticum turgidum*, *Oryza sativa*, *Vigna unguiculata*, *Medicago truncatula*, and *Zea mays*^51–54^.

miRNA-169 targets *HAP*2, a gene that functions in lots of biotic and abiotic stresses. As the largest miRNA family in *Arabidopsis thaliana*, miRNA-169 has 14 members and can be divided into four groups based on mature miRNA sequences: miRNA-169a, miRNA-169b/c, miRNA-169d/e/f/g and miRNA-169h/i/j/k/l/m/n^55^. Several studies have been carried out to determine whether the different biogenesis in the miRNA-169 family affects the properties of the small RNAs. Combier and coworkers showed that miRNA-169 involves the symbiotic nodule development in *Medicago truncatula*^56^. In general, the expression of miRNA-169 is significantly down-regulated by nitrogen deficiency^57^. It has been shown that transgenic plants are more sensitive to drought stress compared to wild type plants since they exert more overexpression of miRNA-169^58^. Although plants have different signalling pathways for detecting and responding to dehydration shock versus drought stress^59^, the results of this study along with previous investigations show the important role of miRNA-169 in these pathways. As an example, an abscisic acid‐dependent pathway is reported for plants subjected to gradual water stress by withholding irrigation in which, miR169 transcripts are down-regulated during the stress^60^. Furthermore, it has been recently shown that among the plant miRNAs, miRNA-169 is the only miRNA that is inhibited by titanium dioxide nanoparticles with a dose-dependent pattern^61^. During the last two decades, titanium dioxide has become a potentially dangerous contaminant to the environment which undesirably affects the plant growth and development^62^.

One of the conserved miRNA families in plants, the *MIR*3*93* genes, have been found in different plant species^63^. miRNA-393 targets a *TIR*1/*AFB2* auxin receptor^4^ and manipulates the auxin responses^64^, such as controlling the root architecture^65^, regulating leaf development^66^, and maintenance of normal plant growth^67^. It has been found that the overexpression of a cleavage resistant form of *TIR1* leads to an increase in salt tolerance^68^. During the stress, up-regulated miRNA-393 contributes to the repression of auxin signalling by keeping *TIR1* levels low, thereby increasing *AUX/IAA-ARF* heterodimerization^69^. Besides, miRNA-160 and miRNA-167, which are also up-regulated as a result of the stress, down-regulate *ARF* levels and consequently, *ARF* mediated gene expression. Therefore, overall *ARF*-mediated gene expression is suppressed by miRNA-393, miRNA-160 and miRNA-167, leading to the attenuation of plant growth and development under stress, and possibly promoting plant stress tolerance as well.

Finally, miRNA-396, as an important contributor to plant stress, targets four classes of stress resistance protein: pathogen-related, nucleotide binding site resistance protein-like, dirigent-like, and ribonuclease-like proteins^70^. Growth regulating factors targeted by miRNA-396 are cell cycle regulators, which control plant growth and differentiation^71^. miRNA-396 contributes to leaf and flower shape control and axillary meristem maintenance as it balances differentiation and proliferation of cell masses and hence morphogenesis. Stress induction of miRNA-396 represses cell multiplication under unfavourable conditions^20^.

### Supervised prediction of plant stress by having the plant miRNAs’ concentration

The results of the contribution of miRNAs to the plant stress response revealed that stress-involved miRNAs were up-regulated or down-regulated towards all the studied stresses which means that the behaviour of these miRNAs is not specific to the stress. This non-specificity limits the performance of predicting the plant stress by having the concentration of one single miRNA even with the most sophisticated computing tools. Some of the research studies have shown that there are several miRNAs that function as specific indicators of plant biotic and abiotic stresses in some plants^72^. However, in the present study, the concentration of the miRNAs did not exert a selective and specific change towards the stresses.

To untie this knot and to introduce a reliable method for the prediction of plant stress by having the plant miRNAs’ concentration, the most important contributing miRNAs in plant stresses which were obtained by the feature selection algorithm in the previous subsection were considered to train the machine learning algorithms. Therefore, the concentration of miRNA-169, miRNA-393, and miRNA-396 in different stress levels were used to train several machines including DT, SVM, LSSVM, and NB. The detail, types, and parameters of these regression-based machine learning algorithms are brought in Methods. The reason why I emphasis on the term “regression” is that in contrast with the feature selection method used in this study, the outputs of machine learning algorithms are continuous (and not discrete). As an example, for a sample in which the concentration of miRNA-169, miRNA-393, and miRNA-396 are 283, 213, and 1307 fM, respectively, a well-trained machine should have an output equal to 80 for the salinity stress which shows that the sample has irrigated with water containing 80 mM of NaCl (see Table 1). Predicting values lower or higher than 80 shows the undesirability of the machine and *R*^2^ of the machine decreases. It should be noted that the machines predict the type of the stress (i.e., salinity, drought, heat, and cold) along with the stress level. In this situation, the researcher does not need to know the type of stress before predicting the stress level by having the miRNA concentrations using the machines since both the type and level of the stress are predicted by the machines simultaneously. Results showed that all the machines were capable of accurate prediction of the stress type. However, some machines were more accurate in predicting the stress level while some of them were less accurate.

The results of model performance evaluation in the prediction of plant stress is shown in Table 3. According to the table, the performance of SVM was better than the performance of other machine learning methods in prediction of plant stress. SVM was able to predict the output with *R*^2^ = 0.96 which means that if we measure the concentrations of miRNA-169, miRNA-393, and miRNA-396 in *Arabidopsis thaliana* plant leaf, there is a good chance that we will be able to predict the plant stress using SVM. It should be noted that in this study and possible similar investigations in the future including the relationships between miRNA concentrations and plant stress, it is not possible to use artificial neural networks (ANNs) as a learning algorithm since ANNs require a lot of training data for the optimization of sigmoid functions belonging to the hidden layer’s neurons^73^. Therefore, in this study, where the number of training samples was small, the optimization process cannot be properly carried out even by using back-propagation algorithms. Furthermore, this small number of training data may result in over-fitting and local minima in ANNs. These phenomena can cause an unrealistic increase in the *R*^2^ of the model.

**Table 3:** Performance evaluation of regression models in the prediction of plant stress.

Model	Parameters	*R*^2^
DT	type = ID3	0.85
	type = C4.5	0.79
	type = CART	0.89
	type = CHAID	0.88
	type = MARS	0.64
SVM	kernel type = linear, *γ* = 0.01	0.76
	kernel type = linear, *γ* = 0.05	0.77
	kernel type = linear, *γ* = 0.10	0.72
	kernel type = polynomial, *γ* = 0.01	0.80
	kernel type = polynomial, *γ* = 0.05	0.83
	kernel type = polynomial, *γ* = 0.10	0.82
	kernel type = Gaussian, *γ* = 0.01	0.87
	kernel type = Gaussian, *γ* = 0.05	0.96
	kernel type = Gaussian, *γ* = 0.10	0.92
	kernel type = sigmoid, *γ* = 0.01	0.86
	kernel type = sigmoid, *γ* = 0.05	0.88
	kernel type = sigmoid, *γ* = 0.10	0.80
LSSVM	kernel type = linear, *γ* = 0.01	0.72
	kernel type = linear, *γ* = 0.05	0.81
	kernel type = linear, *γ* = 0.10	0.81
	kernel type = polynomial, *γ* = 0.01	0.79
	kernel type = polynomial, *γ* = 0.05	0.80
	kernel type = polynomial, *γ* = 0.10	0.82
	kernel type = Gaussian, *γ* = 0.01	0.87
	kernel type = Gaussian, *γ* = 0.05	0.90
	kernel type = Gaussian, *γ* = 0.10	0.83
	kernel type = sigmoid, *γ* = 0.01	0.74
	kernel type = sigmoid, *γ* = 0.05	0.79
	kernel type = sigmoid, *γ* = 0.10	0.71
NB	—	0.63

A comparison among machine learning results shows that CART and CHAID had better performance compared to other DT algorithms (Table 3). Results of SVM and LSSVM methods showed that Gaussian kernel was more accurate than other kernels. The suitable kernel used in SVM method depends on the type of samples’ scattering in the feature space. Former studies have shown that Gaussian kernel is useful in modelling many biological and biotechnological phenomena^74^. NB is another learning algorithm which is based on logistic regression. Logistic regression is mainly used in cases where the output can be expressed as Boolean values. Table 3 shows that this type of model did not have acceptable results.

## Conclusions

This study is the first report of using machine learning to investigate the contribution of miRNAs in plant stress response. Although the response of *Arabidopsis thaliana* miRNAs towards abiotic stresses such as drought, salinity, cold, and heat is not specific, machine learning can be a useful technique to predict plant stress by having the concentration of stress-involved miRNAs. To do this, laboratory data of miRNA concentrations in different levels of plant stress were extracted using an optical nanoparticles-based biosensor to construct a database required by the machine learning algorithms. Then, feature selection algorithm showed that miRNA-169, miRNA-159, miRNA-396, and miRNA-393 have the highest contributions to plant response towards drought, salinity, cold, and heat, respectively. Furthermore, miRNA-169, miRNA-393, and miRNA-396 were considered as the input variables of machine learning algorithms to predict plant stress since they had the highest contributions in all the studied stresses. The results of this study confirm the hypothesis describing machine learning as an efficient technique to improve our knowledge about the relationships between plant stress and miRNAs’ expression.

## Methods

### Plant materials and growth condition

Double-knockout mutant (*acl*5*/spms*) seeds of *Arabidopsis thaliana* ecotype Columbia (Col-0) were surface-sterilized by treating with 70% ethanol for 5 min, then in a solution of 1% sodium hypochloride and 0.1% Tween 20 for 15 min, followed by extensive washing with sterile distilled water. Seeds were then sown in moistened peat pellets, stratified at 4 °C for 2 d, and then transferred to a growth room. Macro and micro nutrient fertilization management of the plants was according to Cipollini^75^. The relative humidity and temperature of the growth room was adjusted at 70 ± 5% and 22 °C under a light intensity of 100 μmol m^−2^ s^−1^ with a photocycle of 16/8 h (light/dark). Complete and equally irrigation of all plants was conducted with 100% of field capacity.

### Stress treatments

Twenty-five-day-old (sixth true leaf stage) seedlings were used for stress investigations. Control seedlings were kept in the condition described above. Each stress was conducted individually at four levels with five replications for 15 days.

Drought treated plants were stressed by withholding water until soil water potential became different with field capacity^76^. Soil moisture was measured daily by a time-domain reflectometry (TDR) device (PMS-714, LUTRON, Taiwan). The soil moisture level was maintained at a level that was nonlethal and above the wilting point, at 85% (*W*_1_), 70% (*W*_2_), 55% (*W*_3_), and 40% (*W*_4_) of field capacity to study different severities of drought stress. Salinity treated plants were irrigated by water which contained different concentrations of NaCl, i.e., 20 mM (*S*_1_), 40 mM (*S*_2_), 60 mM (*S*_3_), and 80 mM (*S*_4_). Higher concentrations of NaCl, more than 100 mM, may result in lethal damage to the young plants^77^. For cold treatment, the temperature of growth room was adjusted at 16 °C (*C*_1_), 12 °C (*C*_2_), 8 °C (*C*_3_), and 4 °C (*C*_4_) whilst seedlings of the same growth stage were kept at 28 °C (*H*_1_), 32 °C (*H*_2_), 36 °C (*H*_3_), and 40 °C (*H*_4_) for heat treatment. The threshold values to select the stress temperatures was according to Kaplan and coworkers^78^.

### miRNA concentration determination

Total RNA was isolated from 50 mg of the uppermost leaf of forty-day-old plants using Total RNA Purification Kit (Norgen Biotek, ON, Canada) according to the manufacturer’s instructions based on Yamaguchi and coworkers^79^. The concentration of plant stress-involved miRNAs in the isolated samples was measured using a gold nanoparticles (AuNPs)-based biosensor. The list of the miRNAs and their sequence is brought in Table S1 in Supporting information. Biosensor preparation was carried out in three steps according to Hakimian and coworkers^39^ and Asefpour Vakilian^80^ which is briefly described below:

Step 1: 100 μL of polyethylenimine (PEI) (42 mM) was added to 3 mL of HAuCl_4_ (1.5 M) under vigorous stirring at constant pH of 7.4 which was adjusted by adding HCl to the solution. Afterwards, the temperature of solution was increased so its colour changed from yellow to red as an indication of the reduction process^39^. PEI-AuNPs were then dialyzed against deionized water using a 3.5 kDa molecular weight cut-off membrane. The resulting red solution was stored at 4 °C before use. Five microliters of the sample were incubated with 40 μL of synthesized PEI-AuNP for 30 min at room temperature.

Step 2: 1.5 mL of sodium citrate 1% was added to 21 mL of boiling HAuCl_4_ solution (0.8 mM), whilst vigorously stirring until its colour changed from pale yellow to deep red. The solution, was then stirred for an additional 15 min and gradually cooled down to room temperature. After that, 400 μL of the solution was mixed with 2 μL of Tween-20 and 400 μL of each thiolated probe (1 μM). Probes and their sequence are brought in Table S2 in Supporting information. The product was left for 48 h in room temperature and then, centrifuged for 23 min at 10,000 rpm. Finally, the supernatant was removed and the oily red precipitate re-dispersed in 200 μL of deionized water.

Step 3: by mixing 5 μL of the products from the steps 1 and 2, probe-target hybridization resulted in the reduction of distance between nanoparticles and an interparticle cross-linking aggregation happened. The higher the target concentration, the greater the aggregation is. As an indicator of reaction, the colour of mixture changed from red-pink to pink and the absorption intensity decreases at 530 nm^39^. Since the UV-Vis absorptions at 530 and 750 nm indicate the quantity of dispersed and aggregated AuNPs, respectively^81^, the absorbance ratio of 750/530 nm was considered as an indicator of probe-target hybridization, and consequently, the concentration of target miRNA. In this study, UV-Vis absorption spectra of the aggregated particles were recorded after 15 min of reaction using a multi-mode microplate reader (SpectraMax iD5, Molecular Devices, USA).

### Statistical analysis

The data obtained from each stress source investigation were individually subjected to analysis of variance (ANOVA) using LSD test at the significance level of 0.01 in SAS 9.0 programming environment.

### Database preparation

The measured concentrations of the studied miRNAs at different stress levels were used to construct a database suitable for machine learning purposes. Due to the fact that statistical analysis showed that replication does not have significant effects on the results, mean values were used for database preparation. Since the effects of four levels of four stress conditions were studied on the concentration of 11 stress-involved miRNAs, a total of 4 × 4 patterns along with one control pattern were used to construct the database. Each pattern consisted of the plant stress level and the corresponding miRNA concentrations.

### Feature selection algorithms

Since one of the research objectives was to investigate the contribution of miRNAs to the plant response towards studied abiotic stresses, miRNA concentrations were considered as the inputs of information theory-based feature selection algorithms whilst stress levels as discrete classes were considered as outputs. Cooperative game theory was used to score the miRNAs based on their contributions to the plant stress response. Intrinsic correlative structures among variables results in different importance of every individual. Cooperative game theory focuses on evaluating the importance (in other words, power) of each feature (input) using the Banzhaf power index^32,82,83^.

In brief, the Banzhaf index can be described as follows^32^: A winning coalition is one for which *v*(*S*) = 1 and a losing coalition is one for which *v*(*S*) = 0. Each coalition *S*U{*i*} that wins when *S* loses is called a swing for player *i*, because the membership of player *i* in the coalition is crucial to the coalition winning. Let *σ*_*i*_(*N*,*v*) be the number of swings for *i*, and let *σ*_o_(*N*,*v*) be the total number of swings of all players in the game. Then, the normalized Banzhaf index is *b*_*i*_(*N*,v) = *σ*_*i*_(*N*,*v*)/ *σ*_*o*_(*N*,*v*). The idea is that every subset of features can be regarded as a candidate subset for the final selected optimal subset^32^. Thus, the power of each feature can be measured by averaging the contributions that it makes to each of the subset which it belongs to. The algorithm of the feature selection method was implemented using a code written in MATLAB R2016b programming environment (Mathworks, MA, USA).

### Learning-based regression models

Although statistical regression models provide reliable mathematical equations to calculate the dependent variable by having the input features, the number of input features should not generally exceed 2 since finding model parameters is rather difficult in high-dimension problems^74,84^. In contrast, machine learning methods can learn a database including hundreds of input features and corresponding dependent variables^85^. In this study, learning-based regression models including DT, SVM, LSSVM, and NB were used to predict plant stress by having the miRNA concentrations as inputs. Since the number of patterns in this study is limited, node-based algorithms, e.g., artificial neural networks (ANN) seem to be inefficient in modelling the database and therefore, they were not used.

Different types of DT modelling including iterative dichotomiser 3 (ID3), statistical model (C4.5), classification and regression tree (CART), chi-squared automatic interaction detection (CHAID), and multivariate adaptive regression splines (MARS) were used in the modelling. SVM and LSSVM models have two parameters including kernel type and kernel parameter which affect the performance of the model^86^. Three kernel types including linear (*f = γ xx*_*o*_), polynomial (*f = *(*γ xx*_*o*_)^3^), Gaussian (*f* = exp(−*γ* (*x−x*_*o*_)^2^)) and sigmoid (*f* = tanh(*γ xx*_*o*_)) were considered for modelling where *f* is the kernel function, *γ* is the kernel parameter, *x* is a train or test sample in the modelling hyperplane, and *x*_*o*_ is the origin point in the hyperplane^87^. The machine learning methods were implemented using a code written in MATLAB R2016b programming environment (Mathworks, MA, USA).

To investigate the performance of the machine learning methods, 3-fold cross-validation was used for training and testing. The performance of the models was evaluated based on the coefficient of determination (*R*^2^). The higher the *R*^2^, the better performance of the machine learning model is.

## Supplementary information

Supplementary Data.
