# Supplementary material for: Machine learning improves our knowledge about miRNA functions towards plant abiotic stresses
Source: Sci Rep. 2020 Feb 20;10:3041. doi: 10.1038/s41598-020-59981-6 (PMC7033123; doi:10.1038/s41598-020-59981-6)
Supplement: Supplementary file 1 — Supplementary Data. [file 41598_2020_59981_MOESM1_ESM.pdf]

Supporting information

Machine learning improves our knowledge about miRNA functions towards plant abiotic stresses

Keyvan Asefpour Vakilian[1,2]

[1] Department of Agrotechnology, College of Abouraihan, University of Tehran, Tehran, Iran

[2] Private Laboratory of Biosensor Applications, Hamadan, Iran

Email address for correspondence: keyvan.asefpour@ut.ac.ir

ORCID: 0000-0001-5035-7727

Table S1. Studied miRNAs and their sequence

miRNA	Sequence
miRNA-156	5′-UGACAGAAGAGAGUGAGCAC-3′
miRNA-159	5′-UUUGGAUUGAAGGGAGCUCUA-3′
miRNA-167	5′-UGAAGCUGCCAGCAUGAUCUA-3′
miRNA-168	5′-UCGCUUGGUGCAGGUCGGGAA-3′
miRNA-169	5′-CAGCCAAGGAUGACUUGCCGA-3′
miRNA-170	5′-UAUUGGCCUGGUUCACUCAGA-3′
miRNA-171	5′-UGAUUGAGCCGCGCCAAUAUC-3′
miRNA-319	5′-UUGGACUGAAGGGAGCUCCCU-3′
miRNA-393	5′-UCCAAAGGGAUCGCAUUGAUCC-3′
miRNA-396	5′-UUCCACAGCUUUCUUGAACUG-3′
miRNA-398	5′-UGUGUUCUCAGGUCACCCCUU-3′

Table S2. Designed probes for the studied miRNAs

miRNA	Probe sequence
miRNA-156	5′-AAAAAAAAAAGTGCTCACTCTCTTCTGTCATTTTTTTTTT-HS-3′
miRNA-159	5′-AAAAAAAAAATAGAGCTCCCTTCAATCCAAATTTTTTTTTT-HS-3′
miRNA-167	5′-AAAAAAAAAATAGATCATGCTGGCAGCTTCATTTTTTTTTT-HS-3′
miRNA-168	5′-AAAAAAAAAATTCCCGACCTGCACCAAGCGATTTTTTTTTT-HS-3′
miRNA-169	5′-AAAAAAAAAATCGGCAAGTCATCCTTGGCTGTTTTTTTTTT-HS-3′
miRNA-170	5′-AAAAAAAAAATCTGAGTGAACCAGGCCAATATTTTTTTTTT-HS-3′
miRNA-171	5′-AAAAAAAAAAGATATTGGCGCGGCTCAATCATTTTTTTTTT-HS-3′
miRNA-319	5′-AAAAAAAAAAAGGGAGCTCCCTTCAGTCCAATTTTTTTTTT-HS-3′
miRNA-393	5′-AAAAAAAAAAGGATCAATGCGATCCCTTTGGATTTTTTTTTT-HS-3′
miRNA-396	5′-AAAAAAAAAACAGTTCAAGAAAGCTGTGGAATTTTTTTTTT-HS-3′
miRNA-398	5′-AAAAAAAAAAAAGGGGTGACCTGAGAACACATTTTTTTTTT-HS-3′
